# Detection of inorganic arsenic in rice using a field-deployable method with Cola extraction

**DOI:** 10.1007/s00216-023-05041-7

**Published:** 2023-11-23

**Authors:** Silvia Wehmeier, Marc Preihs, Julia Dressler, Andrea Raab, Jörg Feldmann

**Affiliations:** 1 Institute of Chemistry, Analytical Chemistry, University of Graz, Graz, Austria; 2 Institute of Inorganic and Analytical Chemistry, Westfaelische Wilhelms-University Muenster, Munster, Germany

**Keywords:** Inorganic arsenic, Rice, Cola extraction, Field method kit, Gutzeit, HPLC-ICP-MS

## Abstract

Rice is a staple food and known to accumulate inorganic arsenic (iAs), which is a class 1 carcinogen to humans. Arsenic field-deployable method kits, designed for water testing, are able to screen iAs in rice, to assure food safety and quick decision-making without the need for laboratory analysis. For the arsenic extraction within the field method, nitric acid is used. To make the field method on-site safer, cost-effective and easier to handle, the method was adapted using a Cola in the extraction process. The adapted field-deployable method was tested by screening a total of 30 rice and rice products from the Austrian market. To verify the results obtained by the Cola extraction field-deployable method, the obtained iAs concentration was compared to HPLC-ICP-MS results. The Cola extraction field method obtained an LOD of 39 µg iAs kg^−1^ rice, and with an average reproducibility of 14% RSD, the method was capable of recording no false-negative but 7% false-positive values at the 2023 updated European Commission (EC) limits for rice. All, but one, screened rice samples were within the EU limits for iAs in rice and rice products.

**Supplementary information:**

The online version contains supplementary material available at 10.1007/s00216-023-05041-7.

## Introduction

Inorganic arsenic (iAs; sum of arsenite and arsenate), a class I carcinogen, received a maximum contaminant level (MCL) in rice and rice-based commodities, which was implemented in an EU regulation in 2016 and updated in 2023 [1]. Here, also a complex analytical method using HPLC coupled to ICP-MS for arsenic speciation has been recommended to monitor iAs in those commodities. There are however disadvantages for this methodology. It needs a sophisticated infrastructure with stable electricity and argon supply as well as a large investment on instrumentation and well-trained personnel.

In 2003, the World Health Organization Special Programme for Research and Training in Tropical Diseases (WHO/TDR) published criteria for monitoring biomarkers for tropical diseases summarized in the acronym ASSURED (affordable, sensitive, specific, user-friendly, rapid, equipment-free, delivered) to meet the needs for the developing world. This concept can be extended for monitoring of environmental issues and food quality, e.g. for iAs in rice.

We have developed a field-deployable method for iAs in rice as a screening method based on the Gutzeit method used for arsenic in water [2]. In this method, the milled rice grain was extracted by 1% nitric acid. Afterwards, borohydride was added, and volatile arsine (AsH_3_) was generated which reacted with a soaked HgBr_2_ solution to form a yellow to red complex. This 1-h process revealed semi-quantitative data for iAs in rice. The method was verified by comparison with the results from the HPLC-ICP-MS [2]. The method was tested afterwards in the agricultural fields of Malawi [3] to gain an immediate response about the iAs in the local rice. It became clear that the bottleneck of this field method is the use of expensive and not readily available nitric acid for extraction.

The aim of this study was to replace nitric acid with a cheap worldwide standardized extractant which can be purchased everywhere in the developing world. Cola a phosphoric acid-containing fizzy drink can be bought everywhere worldwide. Here, we studied an extraction with Cola with subsequent use of the field-deployable method for iAs in rice and compared the results with the use of an HPLC-ICP-MS method, which was validated with the use of a CRM rice to close the metrology chain.

## Materials and method

### Samples

Thirty rice samples of different brands and varieties, including infant rice products, were purchased in a variety of food shops around Graz in Austria. The aim was to cover a range of samples (Table S1) as a proof of concept for the Cola extraction method using the field-deployable method. Samples were prepared for total As and speciation analysis as follows. All rice samples (at least half or more of a consumer package) were homogenized with a Retsch ZM 300 Mill (Retsch GmbH, Haan, Germany).

### Sample preparation for total As (tAs) analysis

Two hundred mg of milled rice (1 mm) (ZR2000, Retsch GmbH, Haan, Germany) (weighed to 0.1 mg) was digested in 2 mL ultra-pure water (18 M cm; Merck Millipore, Darmstadt, Germany) and 3 mL of sub-boiled (Savillex, Eden Prairie, USA) nitric acid Rotipuran p.a. ≥ 65% (Carl Roth, Karlsruhe, Germany) using quartz vessels and an UltraClave (Milestone Srl, Sorisole, Italy). The digestion programme was 10 min ramp to 80 °C, 15 min ramp to 150 °C, 20 min ramp to 250 °C and 250 °C for 30 min. After digestion, the samples were diluted with ultra-pure water to 30 mL and stored at room temperature until ICP-MS analysis. The same preparation was done for the certified reference standard rice flour NIST1568b.

### Total As analysis in rice

As was determined in the digest with ICP-MS (7900 Agilent) in collision cell mode with helium as the collision gas and argon as the carrier gas (Table S2). For As, the mass to charge (*m*/*z*) ratio of 75 was measured. Additionally, an internal standard was added via T-piece containing 200 mg L^−1^ Ge (*m*/*z* 74) and In (*m*/*z* 115) each. The calibration ranged from 0.01 µg to 10 µg As L^−1^ in 10% HNO_3_, using a 1000 mg L^−1^ As standard (Carl Roth GmbH, Karlsruhe, Germany) (Table 1).

### Sample preparation for speciation analysis

For the extraction, 10 mL of extraction solution (1% v/v nitric acid, 1% v/v H_2_O_2_) were added to 100 mg (weighted to 0.1 mg) of milled rice. The samples were placed in a water bath (GFL 1083, Lauda Dr. R. Wobser GMBH & Co. KG, Lauda-Königshafen, Germany) at 99 °C for 1 h with light shaking. Afterwards, the extracts were centrifuged at 4000 rpm (Rotina 420 R, Hettich GmbH, Tuttlingen, Germany) for 15 min. The supernatant was then filtered with 0.22-µm filters (BGB Analytik GmbH, Austria).

### Arsenic speciation

Speciation analysis was carried out on an Agilent HPLC 1260 Infinity system coupled to an Agilent 7700 ICP-MS. Species separation was achieved using a Hamilton PRP-X100 anion exchange column (dimensions, 10 μm; 4.1 × 250 mm) with 40-mM ammonium carbonate aqueous buffer (pH 9.2) as mobile phase (flow rate, 1 mL min^−1^), 50 μL sample injection volume. The column was directly connected to the ICP-MS nebulizer via a short length of PEEK-tubing. The mass to charge (*m*/*z*) ratios Cr 53, As 75, Se 77 and 82 were selected for detection in no gas mode (Table S3). For speciation analysis, mixed standards with dimethyl arsenic acid (DMA), methylarsonic acid (MMA) and inorganic arsenic as arsenate were prepared in 1% H_2_O_2_ from 1 g As L^−1^ stock solution (Merck). To determine the extraction efficiency (EE) and chromatographic recovery (CR), the extracts were remeasured in 10% HNO_3_ on ICP-MS with an additional gas (option gas CO_2_/Ar; 17%; nebulizer gas flow 0.89 mL min^−1^) as shown in Table 2.

### Sample preparation for the field method kit

Milled rice (1 mm; 5 g per replicate; weighed to 0.01 g) was extracted in 50 mL Coca Cola (Classic) (other Cola products showed less reproducibility presumably due to the different behaviour in the foam formation) under light stirring by boiling the mixture at around 90–100 °C temperature for 15–20 min using an electric stove with adjustable control knob. Thereafter, 50 mL of deionized water were added, and the mixture was filtered with a sieve (0.1 mm) before analysis.

### iAs determination with the field method kit

The deployable field method kit (Palintest; Gateshead, United Kingdom) was used to determine the iAs in the Cola extracted rice. Such field method is based on the Gutzeit reaction [2, 4], in which the sample containing As(III) and/or As(V) reacts with sodium borohydride under acidic conditions and converts both species of iAs to arsine gas (AsH_3_). For each sample, the field method kit was zeroed with a blank filter paper. Seventy-five microlitre sieved rice extract was placed in the reaction vessel; after the addition of sulfamic acid (98%; Sigma-Aldrich), 4 drops of Antifoam B emulsion (Sigma-Aldrich), the reaction was started with a NaBH_4_ tablet. After the tablet was completely dissolved, the iAs concentration was determined using the digital detector. Prior, the detector was calibrated to adjust for the 75 mL sample volume, instead of the 50 mL volume of the field method kit.

For the calibration, a blank rice was extracted with Cola (as described above) and spiked with volumes of 1000 mg L^−1^ As(V) (Carl Roth GmbH, Karlsruhe, Germany) in 1% (v/v) HNO_3_ solutions to achieve a calibration range from (5 to 50) µg iAs L^−1^ extracted rice matrix. The limit of detection (LOD) and limit of quantification (LOQ) were determined using the calibration method (DIN 32645) [5].

**Table 1:** Validation of the reference method using 1% v/v nitric acid and 1% v/v H_2_O_2_ extraction with subsequent HPLC-ICP-MS detection. EE (extraction efficiency) was 97%; CR (chromatographic recovery) was 95% and total digestion (recovery 105%). Error is given as SD (*n* = 3)

	DMA µg As kg^−1^	MMA µg As kg^−1^	iAs µg As kg^−1^	Total As µg As kg^−1^
LOD	9	0.4	8	41
SRM NIST 1568b (measured)	169 ± 7 (4%)	11 ± 1 (6%)	93 ± 1 (1%)	299 ± 5 (2%)
SRM NIST 1568b (certified)	180 ± 12	12 ± 4	92 ± 10	285 ± 14

## Results and discussion

**Fig. 1:**
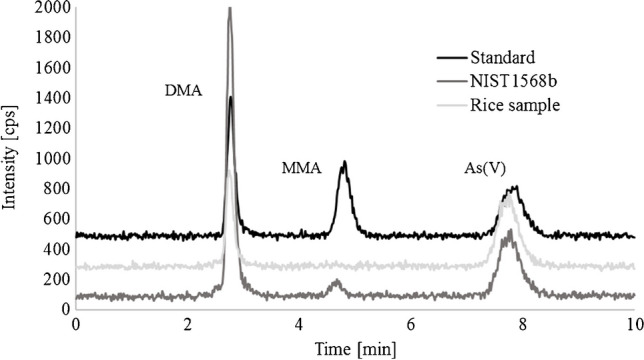
HPLC-ICP-MS chromatogram of standard solution containing DMA, MMA and arsenate (As(V), a CRM (NIST1568b) and a rice sample after nitric acid extraction (detection m/z 75 for As). Individual chromatograms are shown with an offset

To characterize the reliability of the Cola extraction approach with the field method, a comparison to a reliable reference method is needed. Here, we analysed a certified reference material (NIST 1568b) using 1% v/v nitric acid and 1% v/v H_2_O_2_ extraction with subsequent HPLC-ICP-MS analysis. The total arsenic concentration in the rice and rice products was determined by full acid digest and ICP-MS analysis. The analysed reference material NIST 1568b Rice Flour obtained a concentration of (299 ± 5) μg tAs kg^−1^. This value is in agreement with the certified value of (285 ± 14) μg tAs kg^−1^. Additionally, iAs, DMA and MMA were also determined, and 93 ± 1 μg iAs kg^−1^, 169 ± 7 μg DMA kg^−1^ and 12 ± 4 μg MMA kg^−1^ were obtained, and the measured As species concentration were within previously reported ranges [6, 7]. A chromatogram is presented in Fig. 1. Data are presented in Table 1. This method is therefore validated for iAs determination in rice and can be used to compare the newly developed analytical method of a Cola extraction with subsequent use of the field kit.

Thirty rice and rice products (a variety of rice for infants, polished and parboiled rice as well as unpolished rice and rice crackers) were analysed for total As and iAs concentration by the reference method. Concentrations of iAs are listed in Table 2.

The total arsenic concentration varied between (89 and 464) µg kg^−1^ rice, with the mean value of 184 µg kg^−1^ rice and a median of 165 µg kg^−1^ rice. The mean relative standard deviation (RSD) was at 6.1%. The iAs varied between (60 and 249) µg kg^−1^ rice. The proportion of iAs from the total As varied over 16–92% with the mean of 74%. The precision of the iAs of the reference method is 3.9% RSD. Dimethylarsinic acid (DMA) was the most abundant organoarsenic species (average of 26%) with a concentration range of 7–350) µg kg^−1^ rice. Only two samples showed a DMA concentration of around 350 µg kg^−1^. Monomethylarsonic acid (MMA) accounted only for 2% of total arsenic, while tetramethylarsonium [8] and the newly identified dimethylarsonylarsinic acid [9] were not detected. Hence, total As in the Austrian market rice gives no reflection on the iAs content of the rice, confirming previous studies [6, 10].

The iAs concentrations were compared with the newly updated EU maximum contaminant level [1] for the respected different rice subgroups (i.e. infant rice 100 µg kg^−1^, polished and parboiled rice 150 µg kg^−1^, unpolished and husked rice 250 µg kg^−1^ and rice cracker 300 µg kg^−1^). It can be established that only 1 out of the 30 samples (unpolished rice) violated the new legislation, which reflects the recent survey [6] of rice on the Austrian market. If the lowest MCL was taken, then 22 out of the 30 could not be sold as rice destined for infants. Therefore, the market rice was a good testing set for the new field-deployable method which contains different type of rice products generating different matrix for the analysis and uses rice samples with iAs at the correct concentration level.

Two previous studies showed the usability, as well as the reproducibility, of the field method for iAs in rice using nitric acid extraction [2, 3]. Before starting with the rice samples, we studied whether the Cola matrix in combination with the rice matrix has an influence on the detection of iAs with the field kit. Low arsenic rice (from Styria, < 0.02 µg kg^−1^) [6] was spiked with arsenate, and a linear range up to 50 µg L^−1^ (Cola extracted rice matrix) was achieved (*R*^2^ = 0.99, Figure S1). The determined concentration for the arsenate standards showed an average variability of 2.4% (*n* = 3). The arsenate spike recovery ranged from 72 to 90% compared to arsenate in water and was comparable to previously reported 1% nitic acid As(V) spiked rice extraction values of 82 to 117% recovery [3]. Therefore, neither the extraction efficiency nor the matrix effect during the arsine production was significantly affected by the Cola matrix. The Cola extraction field method calibration exhibited an LOD of 39 µg iAs kg^−1^ rice showing therefore a similar LODs as previously reported, 50 µg iAs kg^−1^ rice [2] and 47 µg iAs kg^−1^ [3]. Therefore, the Cola extraction field method was able to detect iAs below the EU regulated MCL of 100 μg iAs kg^−1^ [1, 11] rice destined for the production of food for infant and young children.

Results for the 30 rice samples using the Cola extraction field method showed an average iAs concentration of 132 ± 18 μg iAs kg^−1^ rice, with a range of 69–250) μg kg^−1^. The highest concentration 250 ± 40)μg iAs kg^−1^ rice was observed in a long grain rice (R-28). Three samples showed a precision above 40% RSD (Table 2). Low precisions were recorded for three samples (> 40% RSD, see Table 2) which were not very low in arsenic or were of a specific rice produce. An explanation cannot be given. Overall, an acceptable average precision of 14% RSD was achieved.

**Fig. 2:**
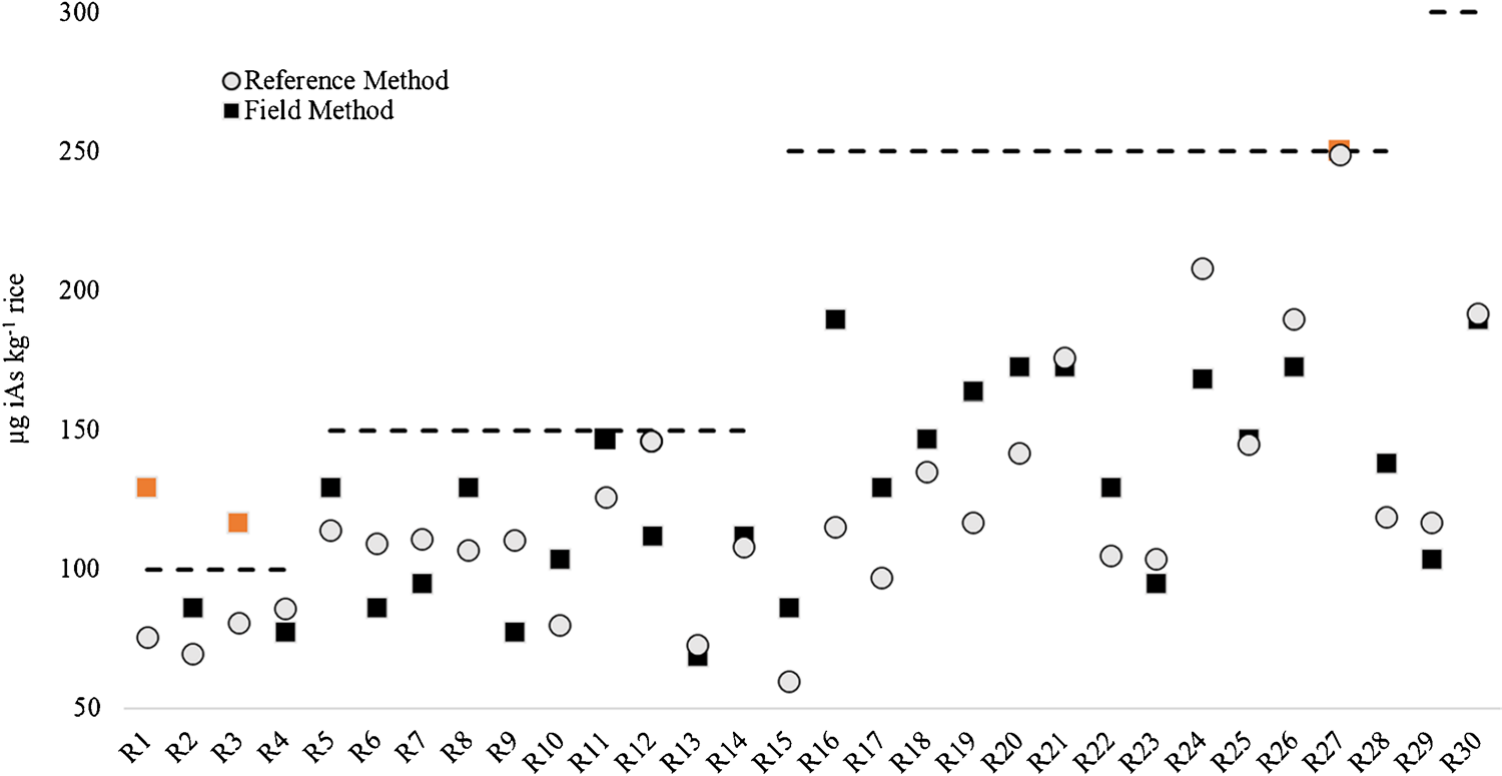
Comparison of the iAs levels of rice samples determined with the Cola extraction field method (■) and HPLC-ICP-MS (o) relative to the 2023 updated EU maximum limit for rice products ((100, 150, 250 and 300) µg iAs kg^−1^). Orange sample points indicate “false-positive” screen by the field method compared to HPLC-ICP-MS

If the rice sample-specific updated MCLs were used as a cut-off criterion, then 3 rice samples were measured to be above the respected MCL; 2 infant rice (100 µg kg^−1^) and 1 unpolished rice (250 µg kg^−1^) (Fig. 2). The unpolished rice was also detected by the reference method. Hence, 2 false-positives were detected with the field-deployable method (7%), and no false-negative was recorded. When the standard error of 14% as precautionary measure was considered and compared to the MCL, the number of false-negative and positive did not increase.

Organoarsenicals (DMA and MMA) were high in one of the samples (80%), while the other 2 rice samples have the average DMA and MMA proportion (together < 30%) from total arsenic. Hence, the reason for the high concentrations measured by the field-deployable method resulting in false-positives is unknown.

**Fig. 3:**
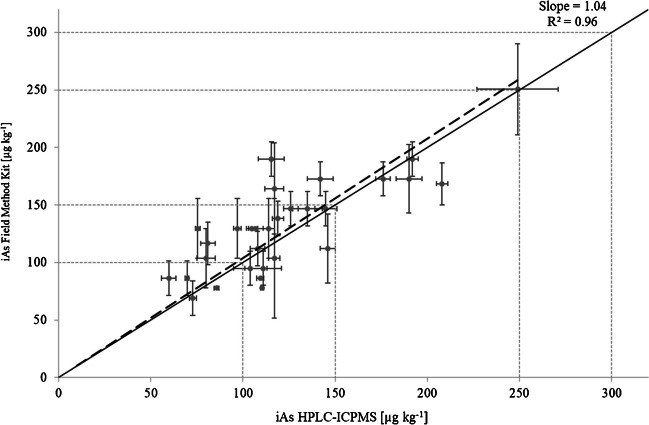
Comparison of the Cola extraction field method to the HPCL-ICP-MS reference method using a linear regression (dashed line). All values (*n* = 30) are given in µg iAs kg^−1^ rice. Error bars shown are SD of three replicates. Vertical and horizontal dotted lines indicate MCL at (100, 150, 250 and 300) μg iAs kg^−1^ for infant food and rice products, for polished rice and husked rice, respectively. Solid black line indicates 1:1 ratio

Overall, this is a very encouraging result, no false-negative means that all samples above or near the MCL would have been identified as high iAs rice. In a step-wise screening, those flagged rice samples with high iAs could then be given to a specialized lab to analyse them with the reference method. The field method can even give semi-quantitative data as shown in Fig. 3. Here, the iAs concentration from the reference method was compared to the iAs concentration achieved by the field method. Although the concentration range was quite small, a good linear correlation (*R*^2^ = 0.96) and a slope of 1.04 showed that the measured concentrations have only a small bias of 4%.

## Conclusion

A simplified field-deployable method to measure iAs in rice was developed using an extraction step with Cola (Coca Cola Classic). This standardized phosphoric acid containing extractant is readily available as fizzy drink all around the world and is cheap. The method did not give false-negative result and only a limited number (7%) of false-positives compared to the validated reference method. Only a limited number of samples show large differences in the comparison with the nitric acid HPLC–ICPMS method, and a limited number of samples showed poor recoveries. This highlights the semi-quantitative nature of this field kit method and should be employed as such. Hence, the field-deployable method is a useful method to screen rice and rice-based commodities in a relative short time in the field. Therefore, this method has all the attributes of the ASSURED method. It is affordable (one sample less than 3 US$), sensitive (below EU MCL for infant rice of 100 µg kg^−1^), specific (no effect from organoarsenicals could be established), user-friendly (no handling of concentrated acids, no well-trained personnel necessary), rapid (within 1 h), equipment-free (only handheld read-outs are necessary) and delivered (can be used in the field without access to electricity). Hence, the Cola extraction can be used in the future to screen rice and rice products on site in rural areas for quick decisions on iAs levels. This approach makes the field method cost-effective and easier as well as safer to use, because Cola is generally widely available and no nitric acid is needed. Therefore, this field-deployable method will empower rice farmers in developing countries to check the iAs in rice for own use or before exporting them to Europe or other parts in the world with import restriction on iAs in rice. Hence, it is in line with the sustainable developing goals (SDGs) of the United Nations.

**Table 2:** Concentration of total and inorganic arsenic in all rice samples (*n* = 30) measured with ICP-MS (tAs) and HPLC-ICP-MS (iAs)

Sample	tAs (µg kg^−1^)^a^	iAs_HPLC-ICP-MS_ (µg kg^−1^)^a^	iAs (%)^b^	iAs_field method_ ( µg kg^−1^)	Recovery (%)^c^
R1	443 ± 28	76 ± 1	19	130 ± 26	172
R2	464 ± 19	70 ± 1	16	86 ± 15	124
R3	119 ± 10	81 ± 4	72	117 ± 18	144
R4	122 ± 10	86 ± 1	69	78 ± 0	91
R5	173 ± 7	114 ± 3	77	130 ± 26	114
R6	162 ± 8	109 ± 2	74	86 ± 0	79
R7	153 ± 8	111 ± 10	80	95 ± 15	86
R8	152 ± 3	107 ± 4	77	130 ± 0	121
R9	167 ± 17	110 ± 1	72	78 ± 0	70
R10	143 ± 12	80 ± 5	61	104 ± 26	130
R11	178 ± 14	126 ± 4	76	147 ± 15	117
R12	175 ± 2	146 ± 4	76	112 ± 30	77
R13	104 ± 4	73 ± 1	70	69 ± 15	95
R14	286 ± 13	108 ± 4	47	112 ± 15	104
R15	89 ± 11	60 ± 4	79	86 ± 15	144
R16	182 ± 11	116 ± 7	80	190 ± 15	165
R17	133 ± 12	97 ± 2	83	130 ± 26	134
R18	185 ± 4	135 ± 8	84	147 ± 15	109
R19	145 ± 15	117 ± 5	84	164 ± 40	140
R20	203 ± 21	142 ± 7	79	173 ± 15	122
R21	210 ± 10	176 ± 4	77	173 ± 15	98
R22	119 ± 7	105 ± 3	81	130 ± 0	123
R23	93 ± 4	104 ± 9	93	95 ± 15	91
R24	218 ± 2	208 ± 3	86	168 ± 18	81
R25	159 ± 4	145 ± 6	85	147 ± 15	101
R26	190 ± 11	190 ± 7	91	173 ± 30	91
R27	262 ± 21	249 ± 22	84	250 ± 40	101
R28	162 ± 7	119 ± 3	68	138 ± 15	116
R29	116 ± 3	117 ± 3	92	104 ± 52	89
R30	218 ± 5	192 ± 3	77	190 ± 15	99

^a^Error is given as SD from three replicates. ^b^% iAs from total As concentration in the rice samples. ^c^(%) recovery of iAs using the Cola extraction with the field method based on iAs measured by HPLC-ICP-MS (iAs from reference method was set to 100%)

### Supplementary information

Below is the link to the electronic supplementary material.

Supplementary file1 (DOCX 46 KB)
