# Supplementary material for: Detection of inorganic arsenic in rice using a field-deployable method with Cola extraction
Source: Anal Bioanal Chem. 2023 Nov 23;416(11):2677–82. doi: 10.1007/s00216-023-05041-7 (PMC11009735; doi:10.1007/s00216-023-05041-7)
Supplement: Supplementary file 1 — Supplementary file1 (DOCX 46 KB) [file 216_2023_5041_MOESM1_ESM.docx]

Supplementary material

Detection of inorganic arsenic in rice using a field method kit: a screening method with Cola extraction

Silvia Wehmeier^1*^, Marc Preihs^1*^, Julia Dressler^1,2^, Andrea Raab^1^, Jörg Feldmann ^1^

**Table S1:** Total As concentrations in rice samples compared to the newly updated MCL for rice and rice-based commodities according to Regulation EC No1881/2006 update 3 March 2023. Deviation is given from standard deviation of three replicates.

| **Sample** | **Brand** | **Rice type** | **Limit [µg iAs kg^-1^ rice]** | **Origin** |
| --- | --- | --- | --- | --- |
| R1 | Alnatura | Rice cakes | 100 | Not given |
| R2 | Beauty Baby | Rice cakes | 100 | Not given |
| R3 | HiPP | Rice cakes | 100 | Not given |
| R4 | Bebivita | Rice cakes | 100 | Not given |
| R5 | Spar | Long grain polished | 150 | Italy |
| R6 | Spar Natur Pur | Risotto rice | 150 | Italy |
| R7 | Bio Gourmet | Pudding rice | 150 | Italy |
| R8 | Spar Premium | Sushi rice | 150 | Italy |
| R9 | Billa | Risotto rice | 150 | Italy |
| R10 | Oryza | Long grain polished | 150 | Belgium |
| R11 | Billa | Long grain parboiled | 250 | Italy |
| R12 | Rapunzel | Puffed rice | 250 | Italy |
| R13 | Bauckhof | Rice flour | 250 | Non EU agriculture |
| R14 | Kellogs | Puffed rice | 250 | Not given |
| R15 | Spar Natur Pur | Basmati unpolished | 250 | India |
| R16 | Spar Natur Pur | Parboiled unpolished | 250 | Italy |
| R17 | Royal Thai | Red rice | 250 | Thailand |
| R18 | Brantner | Round grain unpolished | 250 | India |
| R19 | Brantner | Round grain unpolished | 250 | India |
| R20 | Ben’s Original | Long grain unpolished | 250 | Not given |
| R21 | Nestelberger | Long grain unpolished | 250 | Italy |
| R22 | Nestelberger | Basmati unpolished | 250 | India |
| R23 | Davert | Round grain unpolished | 250 | Italy |
| R24 | Bauckhof | Rice flakes | 250 | EU agriculture |
| R25 | Lotao Deli | Long grain black rice | 250 | EU agriculture |
| R26 | Spielberger | Rice flakes unpolished | 250 | Italy |
| R27 | Rapunzel | Long grain unpolished | 250 | France |
| R28 | Spar Natur Pur | Long grain unpolished | 250 | Italy |
| R29 | Lima | Rice cakes | 300 | EU agriculture |
| R30 | Byodo | Rice cakes | 300 | Italy |

**Table S2** Settings for total As analysis via ICP-MS

| **ICP-MS** |  | **Agilent 7900** |
| --- | --- | --- |
| Scan type |  | Single Quad |
| Gas Mode |  | He |
| RF Power | / W | 1550 |
| Carrier gas flow rate | / L min^-1^ | 1.08 |
| Nebulizer pump | / rps | 0.1 |
| Spray chamber  temperature | / ˚C | 2 |

**Table S3** Settings for speciation analysis (HPLC-ICP-MS for speciation and ICP-MS for total As within speciation analysis)

| **ICP-MS** |  | **Agilent 7700** |
| --- | --- | --- |
| Scan type |  | Single Quad |
| Gas Mode |  | He |
| RF Power | / W | 1550 |
| Carrier gas flow rate | / L min^-1^ | 0.93 |
| Option gas |  | CO_2_/Ar (1 v/v%) |
| Flow rate | / % | 17 |
| Nebulizer pump | / rps | 0.1 |
| Spray chamber  temperature | / ˚C | 2 |

| **HPLC** |  | **Agilent 1260 Infinity** |
| --- | --- | --- |
| Column |  | Hamilton PRP-X100 anion exchange column |
| Mobile Phase |  | 40 mM aqueous ammonium carbonate buffer |
| Injection volume | / µL | 50 |
| Mobile phase flow | / mL min^-1^ | 1 |
| Column temperature | / ˚C | 30 |
| Runtime | / min | 15 |
| **ICP-MS** |  | **Agilent 7700** |
| Scan type |  | Single Quad |
| Gas Mode |  | No gas |
| RF Power | / W | 1600 |
| Carrier gas flow rate | / L min^-1^ | 1.12 |
| Nebulizer pump | / rps | 0.5 |
| Spray chamber  temperature | / ˚C | 2 |

**Table S4** Concentration of total arsenic, DMA and MMA in all rice samples (n = 30) measured with ICP-MS (tAs) and HPLC-ICP-MS (DMA and MMA), as well as their proportion of total arsenic

| **Sample** | **tAs [µg kg^-1^]** | | | **DMA [µg kg^-1^]** | | | | **DMA [%]** | **MMA [µg kg^-1^]** | | | **MMA**  **[%]** |
| --- | --- | --- | --- | --- | --- | --- | --- | --- | --- | --- | --- | --- |
| R1 | 443 | ± | 28 | 326 | ± | 7 | 80 | | 5,2 | ± | 1,6 | 1 |
| R2 | 464 | ± | 19 | 354 | ± | 10 | 83 | | 5,1 | ± | 0,8 | 1 |
| R3 | 119 | ± | 10 | 31 | ± | 1 | 27 | | 1,4 | ± | 0,5 | 1 |
| R4 | 122 | ± | 10 | 37 | ± | 2 | 30 | | 1,4 | ± | 0,5 | 1 |
| R5 | 173 | ± | 7 | 35 | ± | 2 | 23 | | <LOD |  |  | <1 |
| R6 | 162 | ± | 8 | 38 | ± | 3 | 26 | | <LOD |  |  | <1 |
| R7 | 153 | ± | 8 | 26 | ± | 3 | 19 | | 1,7 | ± | 0,4 | 1 |
| R8 | 152 | ± | 3 | 30 | ± | 1 | 22 | | 1,2 | ± | 0,6 | 1 |
| R9 | 167 | ± | 17 | 41 | ± | 2 | 27 | | 1,5 | ± | 0,5 | 1 |
| R10 | 143 | ± | 12 | 50 | ± | 4 | 38 | | 1,9 | ± | 0,9 | 1 |
| R11 | 178 | ± | 14 | 39 | ± | 2 | 24 | | 1,4 | ± | 0,4 | 1 |
| R12 | 175 | ± | 2 | 46 | ± | 2 | 24 | | <LOD |  |  | <1 |
| R13 | 104 | ± | 4 | 31 | ± | 3 | 30 | | <LOD |  |  | <1 |
| R14 | 286 | ± | 13 | 119 | ± | 4 | 52 | | 3,6 | ± | 0,7 | 2 |
| R15 | 89 | ± | 11 | 15 | ± | 2 | 19 | | 1,48 | ± | 0,4 | 2 |
| R16 | 182 | ± | 11 | 29 | ± | 5 | 20 | | <LOD |  |  | <1 |
| R17 | 133 | ± | 12 | 18 | ± | 2 | 15 | | 1,2 | ± | 0,4 | 1 |
| R18 | 185 | ± | 24 | 25 | ± | 1 | 15 | | 1,0 | ± | 0,1 | 1 |
| R19 | 145 | ± | 15 | 20 | ± | 1 | 14 | | 2,3 | ± | 0,6 | 2 |
| R20 | 203 | ± | 21 | 36 | ± | 1 | 20 | | 2,2 | ± | 0,4 | 1 |
| R21 | 210 | ± | 10 | 50 | ± | 2 | 22 | | 2,5 | ± | 0,6 | 1 |
| R22 | 119 | ± | 7 | 25 | ± | 2 | 19 | | <LOD |  |  | <1 |
| R23 | 93 | ± | 4 | 8 | ± | 1 | 7 | | <LOD |  |  | <1 |
| R24 | 218 | ± | 2 | 33 | ± | 1 | 14 | | 2,0 | ± | 0,2 | 1 |
| R25 | 159 | ± | 4 | 25 | ± | 1 | 15 | | <LOD |  |  | <1 |
| R26 | 190 | ± | 11 | 19 | ± | 1 | 9 | | <LOD |  |  | <1 |
| R27 | 262 | ± | 21 | 43 | ± | 3 | 14 | | 4,9 | ± | 0,6 | 2 |
| R28 | 162 | ± | 7 | 57 | ± | 4 | 32 | | <LOD |  |  | <1 |
| R29 | 116 | ± | 3 | 10 | ± | 1 | 8 | | <LOD |  |  | <1 |
| R30 | 218 | ± | 5 | 56 | ± | 3 | 22 | | 2,8 | ± | 0,5 | 1 |


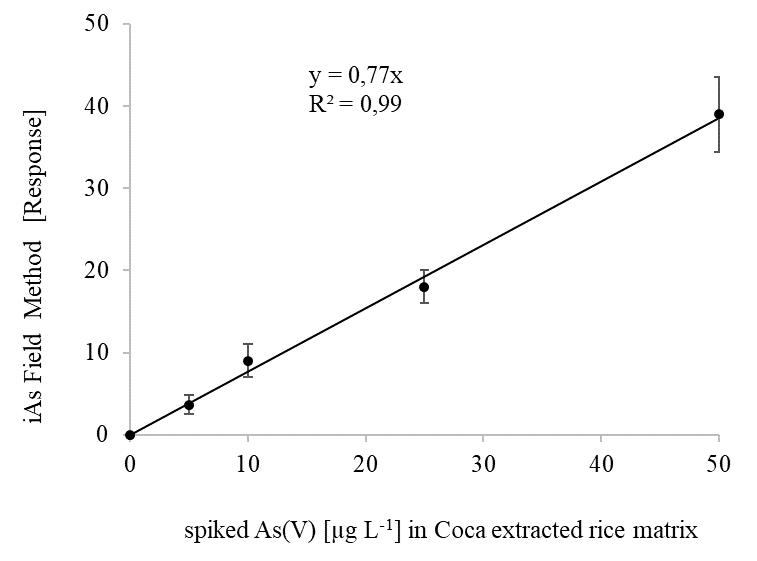


**Fig. S1** Calibration of the field deployable method using Cola extracted blank rice spiked with arsenate
